# A short ^18^F-FDG imaging window triple injection neuroimaging protocol for parametric mapping in PET

**DOI:** 10.1186/s13550-023-01061-7

**Published:** 2024-01-02

**Authors:** Hamed Moradi, Rajat Vashistha, Kieran O’Brien, Amanda Hammond, Viktor Vegh, David Reutens

**Affiliations:** 1 Centre for Advanced Imaging, Australian Institute for Bioengineering and Nanotechnology, The University of Queensland, Brisbane, Australia; 2 ARC Training Centre for Innovation in Biomedical Imaging Technology, The University of Queensland, Brisbane, Australia; 3 Siemens Healthcare Pty Ltd, Melbourne, Australia

**Keywords:** Arterial input function (AIF), Dynamic PET, Parametric mapping, Triple injection protocol, Short window imaging

## Abstract

**Background:**

In parametric PET, kinetic parameters are extracted from dynamic PET images. It is not commonly used in clinical practice because of long scan times and the requirement for an arterial input function (AIF). To address these limitations, we designed an ^18^F-fluorodeoxyglucose (^18^F-FDG) triple injection dynamic PET protocol for brain imaging with a standard field of view PET scanner using a 24-min imaging window and an input function modeled using measurements from a region of interest placed over the left ventricle.

**Methods:**

To test the protocol in 6 healthy participants, we examined the quality of voxel-based maps of kinetic parameters in the brain generated using the two-tissue compartment model and compared estimated parameter values with previously published values. We also utilized data from a 36-min validation imaging window to compare (1) the modeled AIF against the input function measured in the validation window; and (2) the net influx rate ($K_{i}$) computed using parameter estimates from the short imaging window against the net influx rate obtained using Patlak analysis in the validation window.

**Results:**

Compared to the AIF measured in the validation window, the input function estimated from the short imaging window achieved a mean area under the curve error of 9%. The voxel-wise Pearson’s correlation between $K_{i}$ estimates from the short imaging window and the validation imaging window exceeded 0.95.

**Conclusion:**

The proposed 24-min triple injection protocol enables parametric ^18^F-FDG neuroimaging with noninvasive estimation of the AIF from cardiac images using a standard field of view PET scanner.

**Supplementary Information:**

The online version contains supplementary material available at 10.1186/s13550-023-01061-7.

## Background

In clinical practice, diagnostic ^18^F-fluorodeoxyglucose (FDG) positron emission tomography (PET) images are most commonly interpreted by visual inspection, sometimes taking into account information from semiquantitative standardized uptake value (SUV) data [1]. Parametric PET generates images of kinetic parameters describing FDG uptake, estimated from the temporal profile of changes in tissue tracer concentration extracted from dynamic PET data. These kinetic parameters represent the influx of ^18^F-FDG into tissue, $K_{1}$ (ml/g/min), efflux from tissue to blood, $k_{2}$ (1/min), the rates of phosphorylation of ^18^F-FDG and dephosphorylation of ^18^F-FDG-6-phosphate, $k_{3}$ and $k_{4}$ (1/min), respectively, and the net influx rate, $K_{i}$, computed as $K_{i} = K_{1} k_{3} /\left( {k_{2} + k_{3} { }} \right)$ in units of ml/g/min [2]. The parameters can be formulated as unknowns to be estimated in a compartmental model, as originally proposed by Sokoloff et al. in 1977 [3].

Advances in PET technology now enable more detailed analysis of dynamic PET data and parametric PET [4]. These include the development of dynamic image reconstruction algorithms [5], time-of-flight PET data acquisition [6], and whole-body parametric imaging on commercial PET scanners [7]. Long axial field-of-view PET scanners have greatly enhanced PET capabilities in parametric PET [8]. This holds great promise for enhancing disease diagnosis, monitoring treatment responses, and drug development [4, 5, 9–12]. Early studies have shown that combining kinetic parameters and SUV data improves discrimination between benign and malignant lesions, enhances tumor grading accuracy, and provides superior clinical diagnostic information compared to existing SUV-based methods [13–18]. For example, Strauss et al. demonstrated that incorporating ^18^F-FDG PET kinetic parameters with the SUV significantly improved bone lesion classification, particularly for distinguishing grade I from grade III tumors, resulting in an overall diagnostic accuracy of 87.7%, surpassing the 74.7% achieved with SUV alone [16].

Although parametric PET imaging in neurology [19] has been thoroughly researched, it is yet to be routinely implemented in clinical practice. ^18^F-FDG PET parametric imaging shows promise in enhancing the diagnosis and monitoring of brain disorders [4]. For example, Kimura et al. used PET kinetic analysis to differentiate CNS lymphoma from high-grade glioma, emphasizing the value of parameters like $k_{3}$ in diagnosing CNS lymphoma based on distinct glucose metabolism patterns [20]. ^18^F-FDG PET parametric imaging can also assist in monitoring tumor therapy [21]. Nishiyama et al. found that ^18^F-FDG PET kinetic parameters, especially $k_{3}$, were beneficial in the diagnosis and assessment of treatment response of central nervous system lymphoma [22]. FDG uptake parameters correlated with the response to chemotherapy of lymphomas, with significant reductions in both $K_{1}$ and $k_{3}$ being observed after treatment [23]. Parametric PET imaging has also been employed in other contexts in human imaging including the visualization of protein targets in the brain [19], in traumatic brain injury [24, 25] and Alzheimer's disease [26, 27], where it has provided new insights into the underlying changes associated with these neurological conditions.

Despite its potential benefits, parametric mapping is not in wide clinical use [4, 5, 28, 29]. Because current methods require longer scan times that nonparametric imaging, resulting in lower patient throughput and increasing the likelihood of patient discomfort and of movement-related image artifacts that may contribute to erroneous parameter estimates [29–31]. Furthermore, an Arterial Input Function (AIF) is required for parameter estimation. Because arterial blood sampling for the estimation of the AIF is invasive, adds to preparation time, and may have uncommon but serious complications [4, 32], several noninvasive alternatives have been proposed for parametric PET imaging. An appealing, noninvasive alternative to arterial blood sampling is to estimate the AIF directly from PET images. This approach depends on the presence of a suitable artery within the imaging field and has been effectively validated for blood pools such as the heart [33], aorta [34], and femoral arteries [35]. The larger size of these vessels simplifies ROI placement and allows the potential omission of corrections for the partial volume effect [36–40]. Additional minimally invasive approaches involve jointly estimating the AIF and kinetic parameters, with limited blood sampling [41, 42], and the use of a population-derived AIF [43, 44]. Directly estimating the AIF from images mitigates the risk of overfitting found in joint estimation methods. Furthermore, it eliminates the need for blood sampling and, in contrast to using a population-derived AIF, accommodates individual variations in AIF shapes more effectively. In PET brain studies using clinical standard field-of-view scanners and single-bed protocols, accurately estimating the AIF directly from images remains challenging. This is due to the absence of large blood pools in the images and the influence of the partial volume effect, resulting from the vessels' small size in comparison to the limited spatial resolution of PET scanners. A ROI over large blood pools is less prone to partial volume effect than estimates from smaller vascular structures such as the carotid artery [37].

Long axial field of view PET/CT scanners offer the advantage of simultaneous brain imaging and deriving AIF measurements from large vascular structures like the aorta or left ventricle, effectively reducing partial volume effects [40, 45, 46]. However, despite these benefits, the utilization of these scanners is limited due to their small installed base compared to standard field of view scanners, while still requiring hour-long dynamic ^18^F-FDG PET scans from tracer administration.

Recent software advancements and the adoption of multi-pass, multi-bed PET scanning techniques, also known as dynamic whole-body imaging, in standard field of view scanners, hold transformative potential in the field [7, 47]. In dynamic whole-body imaging, encompassing the entire body, it commences with an early cardiac scan to capture the AIF peak. Subsequently, data are collected through multiple whole-body bed passes, enabling kinetic modeling via the linear Patlak analysis [48] to estimate the net uptake rate.

Advancements in whole-body PET/CT scanning have facilitated large clinical studies, as demonstrated in a study assessing 118 lesions in 18 patients, where the combination of $K_{i}$ and SUV imaging enhanced sensitivity and accuracy in detecting malignant lesions while reducing false positives [49]. Another study involving 101 patients demonstrated enhanced lesion contrast and reduced false positives compared to SUV images, providing benefits for specific patient groups and evaluations of treatment responses [11].

In whole-body ^18^F-FDG PET/CT scans, the AIF is usually obtained by placing ROIs over the aorta or left ventricle during an early cardiac scan to capture the AIF peak. Subsequently, AIF shape is sampled through multiple whole-body bed passes [50]. However, it is important to note that precise estimation of individual kinetic parameters like $K_{1}$, $k_{2}$, $k_{3}$ and $k_{4}$ relies on tissue time activity curves obtained from early PET measurements. The current approach in whole-body ^18^F-FDG PET/CT scanning does not allow early tissue activity curve measurement. As a result, this approach primarily facilitates the determination of the net influx rate ($K_{i}$) [51, 52]. Nevertheless, it requires lengthy dynamic ^18^F-FDG PET scans starting from the time of tracer administration.

We aimed to develop a parametric ^18^F-FDG PET brain imaging protocol for standard field of view scanners that allows estimation of individual kinetic parameters in a scan time comparable to that of nonquantitative imaging (24min) and using AIF estimates from a cardiac ROI.

## Theory: triple injection protocol

### Proposed protocol

**Fig. 1:**
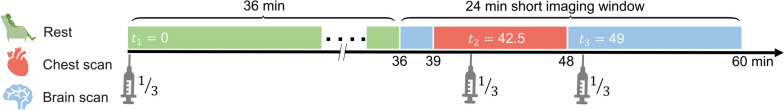
Schematic illustration of the proposed triple injection protocol. Shown are a 1/3 dose initial tracer injection followed by 36 min of rest before dynamic PET data acquisition for 24 min with two additional 1/3 dose tracer injections

The standard parametric ^18^F-FDG PET brain imaging protocol involves a 60-min single-bed position acquisition over the brain after radiotracer injection, with arterial blood sampling to measure the AIF [2]. We propose a triple injection PET imaging protocol (Fig. 1) comprising a 36-min uptake phase outside the scanner and a short 24-min dynamic PET imaging window:

1. The standard radiotracer dosage is divided into three equal doses.
2. The first dose is injected with the patient outside the scanner, followed by an uptake period of 36 min. This allows the late time points of the brain and heart time activity curves to be measured in the subsequent scans.
3. A 3-min dynamic scan of the brain (36-39), comprising two 90 s frames, is then performed.
4. The scanner bed is moved for 9 min of cardiac imaging (39–48 min) comprising, in sequence, 3 × 60 s, 3 × 20 s, 4 × 30 s and 3 × 60 s frames. The second dose of tracer is injected at 42.5 min, during the cardiac scan and the 20 s frames are designed to capture the second peak in vascular activity. The 9-min scan allows us to estimate 3.5 min of the tail of the AIF from the first injection and the initial 5.5 min of the AIF, including its peak, from the second injection.
5. The scanner bed is moved for a 12-min dynamic brain scan (48–60 min), comprising 2 × 90 s and 3 × 180 s frames. The third dose of tracer is injected at 49 min, during the brain scan. This allows measurement of the early portion of the brain time activity profile.

In the next section, we provide the justification and background information underlying this protocol design.

### Rationale

We [52] and others [53–55] have investigated the time windows in ^18^F-FDG dynamic PET acquisitions and AIF measurements that are critical for accurate estimation of kinetic parameters. Several studies have shown that an initial 10–15-min scan, followed by a late 3–10-min scanning window, around 60 min after tracer injection, is sufficient for accurate estimation of kinetic parameters [53–55]. In a previous simulation study, we showed that, using Machine Learning, it is possible to reliably estimate the kinetic parameters using only the first 12 min (0–12 min) and the last 3 min (57–60 min) of the time activity curve (TAC) and AIF [52]. However, the use of two short acquisitions following a single tracer injection dose is not clinically practical because the participant must undergo two separate PET and CT scans separated by a nonscanning period, resulting in a complex workflow and necessitating PET image co-registration. The triple injection protocol allows the acquisition of scans that are early and late with respect to the preceding tracer injection, without an intervening nonscanning period. The model assumes that physiological steady state is maintained from the first injection through to the end of scan acquisition. Furthermore, it assumes that the rate of radiotracer injection remains fairly uniform across three separate manual injections [56]. The total time for the procedure was limited to 60 min to be comparable to standard ^18^F-FDG dynamic PET scanning.

To assess the reliability of estimating the AIF and evaluate the accuracy of kinetic parameter estimation, we conducted a simulation study in MATLAB® R2021b (MathWorks, Natick, MA). We first used 9 min of cardiac imaging to estimate the AIF, and then examined the accuracy of kinetic parameter estimation using a triple injection protocol that totalled 24 min.

### Accuracy of AIF estimation: simulation study

A simulation study was performed using MATLAB® R2021b (MathWorks, Natick, MA) to evaluate the reliability of estimating the AIF using 9 min (3.5  + 5.5 min) of cardiac imaging. We simulated AIFs for three tracer injections of equal dose at times $t_{1}$ = 0, $t_{2}$ = 42.5, and $t_{3}$ = 49 min using a model introduced by Feng et al. [57] and performed a convolution of the model with three impulse functions, each corresponding to a specific injection with a given delay. The resulting triple injection model-based AIF is given by:

1 $$C_{p} \left( t \right) = \frac{1}{3}\left( {\delta \left( {t - t_{1} } \right) + \delta \left( {t - t_{2} } \right) + \delta \left( {t - t_{3} } \right)} \right) \otimes (A_{1} te^{{ - \mu_{1} t}} + A_{2} \left( {e^{{ - \mu_{2} t}} - e^{{ - \mu_{1} t}} } \right)),$$

where $C_{p} \left( t \right)$ is the AIF at time $t$, $\delta$ denotes the Dirac delta function, $t_{1}$, $t_{2}$ and $t_{3}$ are the time delays for each injection in units of min, and $A_{1}$ (kBq/ml), $A_{2}$ (kBq/ml), $\mu_{1}$(1/min) and $\mu_{2}$ (1/min) are the four AIF parameters to be estimated.

**Fig. 2:**
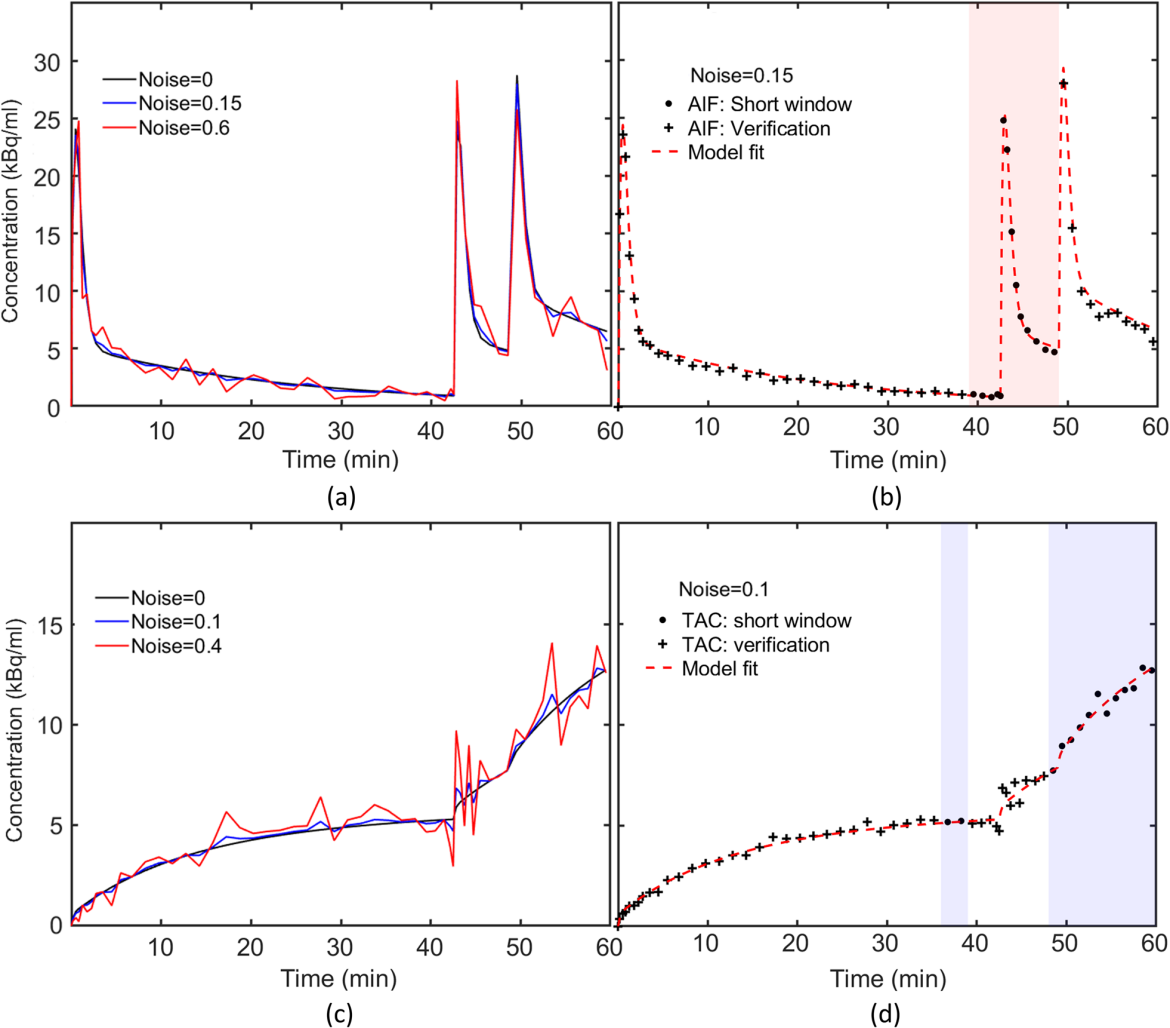
The simulated AIF for parameters $A_{1} = 263 \left( {\text{kBq/ml}} \right)$, $A_{2} = 16 \left( {\text{kBq/ml}} \right)$, $\mu_{1} = 3.56 \left( {1/{\text{min}}} \right)$ and $\mu_{1} = 0.029 \left( {1/{\text{min}}} \right)$ at low (*c* = 0.15) and high noise (*c* = 0.6) levels and the simulated AIF without noise are shown. **b** Example of the simulated and estimated AIF for *c* = 0.15 noise level is shown. Data points denoted by the ‘ + ’ and ‘●’ symbols are measurements taken from LV. Measurements from the 39-48min window (red shaded area) were used to estimate the entire AIF (the red dashed line). **c** Simulated TACs for $K_{1} = 0.075$ (ml/g/min), $k_{2} = 0.15$(1/min), $k_{3} = 0.03$ (1/min), and $v_{b} = 0.03$ at low (*c* = 0.1) and high (*c* = 0.4) noise levels and without noise. **d** Examples of simulated and estimated TACs at *c* = 0.1 noise level. Data points denoted by the ‘ + ’ and ‘●’ symbols are the TAC measurements. The simulated TAC from the 36–39 min and 48–60 min time frames (the blue shaded area) were used for parameter estimation. The simulated TAC corresponding to the ‘ + ’ symbols were used only for validation

The AIF parameters used in the simulation were chosen based on Feng et al.’s published values for mean and standard deviation (SD): $A_{1} = 263 \pm 120{ }$, $A_{2} = 16 \pm 1.32$, $\mu_{1} = 3.56 \pm 1.31{ }$ and $\mu_{1} = 0.029 \pm 0.012$ [57]. We simulated the time course of 81 AIFs over 60 min corresponding to all possible combinations of three values for each parameter—mean, mean + SD and mean—SD. Sequential time frames of 3 × 20 s, 4 × 30 s, 3 × 60 s, 22 × 90 s, 3 × 60 s, 3 × 20 s, 4 × 30 s, 3 × 60s, 2 × 90 s and 3 × 180 s were simulated. Random noise was added to each AIF according to a previously described noise model for PET [58]:

2 $$C_{p}^{\eta } \left( t \right) = C_{p} \left( t \right) + \left( {\eta \left( t \right) \times c \times \sqrt {\frac{{C_{p} \left( t \right)}}{\Delta t}} } \right),$$

where $\eta$ is a pseudo-random number drawn from a Gaussian distribution$,{ }\sim N\left( {0,{ }1} \right)$, $C_{P} \left( t \right)$ is the noise-free AIF, $c$ is a scaling factor to adjust the noise level, $\Delta t$ is the time frame interval in min. Low noise (*c* = 0.15) and high noise (*c* = 0.6) levels were simulated (Fig. 2a) and, for each noise-free AIF and each noise level, 1000 noisy measured AIF time courses were simulated.

AIF parameters were then estimated using 39–48 min of the simulated AIF, corresponding to the period of cardiac scanning in the triple injection protocol. Nonlinear least squares fitting was performed. AIF parameter bounds were set as 0.5 × the minimum and 2 × the maximum of the Feng function parameters used in the simulation. Accuracy of parameter estimation was assessed using:

Area under the curve error (${\text{AUC}}_{{{\text{error}}}}$), which compares the temporal profiles of the simulated and the estimated AIF over the entire 60-min window:

3 $${\text{AUC}}_{{{\text{error}}}} = \frac{1}{R}\mathop \sum \limits_{r = 1}^{R} \frac{{{\text{AUC}}^{r} - \widehat{{{\text{AUC}}^{ } }}}}{{\widehat{{{\text{AUC}}^{ } }}}} \times 100,$$

where ${\text{AUC}}_{{{\text{error}}}}$ is the average percentage error of the area under curve (AUC) of the AIF over R random noise repetitions, and ${\text{AUC}}^{r}$ is the AUC of the estimated AIF and $\widehat{{{\text{AUC}}^{ } }}$ is the AUC of the simulated noiseless AIF.

Normalized root-mean-square error (NRMSE) between the simulated noiseless AIF and the estimated AIF for the entire 60 min using:

4 $${\text{NRMSE}} = \frac{1}{R}\mathop \sum \limits_{r = 1}^{R} \sqrt {\frac{1}{n}\mathop \sum \limits_{i = 1}^{n} \frac{{\left( {f_{i}^{r} - \widehat{{f_{i}^{ } }}} \right)^{2} }}{{\widehat{{f_{i}^{ } }}^{2} }}} ,$$

where $f_{i}^{r}$ denotes estimated AIF values for the $ith$ time point from $rth$ random noise repetitions, and $\widehat{{f_{i}^{ } }}$ is the simulated noiseless AIF and $n$ is the total number of timepoints for AIF estimates.

Figure 1b illustrates the simulated and estimated AIFs for the *c* = 0.15 noise level. At this noise level, the estimated AIF captured the shape of the true AIF. Table 1 lists the mean ${\text{AUC}}_{{{\text{error}}}}$ and NRMSE for both noise levels and for data without noise across the 81 simulated AIFs. The 0.15 noise level data are more relevant for practical implementation of AIF estimation using a cardiac ROI. At this level of noise, the mean ${\text{AUC}}_{{{\text{error}}}}$ was 1.96% and the mean NRMSE was 0.06. AIF estimation error propagates to a similar extent to $K_{1}$ estimation whereas $k_{2}$ and $k_{3}$ estimation are less affected [59]. These findings indicate that the AIF can be accurately estimated using only the 9-min AIF window (39–48 min) of the triple injection protocol described above.

**Table 1:** The mean ${\text{AUC}}_{{{\text{error}}}}$ and NRMSE for two noise levels and for data without noise across the 81 simulated AIFs

Noise level (*c*)	AIF accuracy	
	${\text{AUC}}_{{{\text{error}}}}$(%)	NRMSE
*c* = 0	0.2 ± 0.15	0.04 ± 0.008
*c* = 0.15	1.96 ± 1.49	0.06 ± 0.01
*c* = 0.6	8.21 ± 6.35	0.17 ± 0.08

### Accuracy of kinetic parameter estimation: simulation study

To examine the accuracy of kinetic parameter estimation using the triple injection protocol, TACs were simulated using the irreversible two tissue compartment model (2TCM):

5 $$C_{T} \left( t \right) = \left( {1 - v_{p} } \right)\left( {\left( {\frac{{K_{1} k_{2} }}{{k_{2} + k_{3} }}e^{{ - \left( {k_{2} + k_{3} } \right)t}} + \frac{{K_{1} k_{3} }}{{k_{2} + k_{3} }}} \right) \otimes C_{p} \left( t \right)} \right) + v_{p} C_{p} \left( t \right),$$

where $C_{T} \left( t \right)$ is the measured total tracer concentration in tissue, $C_{p} \left( t \right)$ is the AIF, $t$ is a point in time, $v_{p}$ is the volume fraction of tracer in the plasma pool, and ⨂ denotes the convolution operation.${ }K_{1}$, $k_{2}$, and $k_{3}$ are kinetic parameters defined above.

AIFs were simulated as in the previous simulation study, using the mean AIF parameter values from Feng et al. [57] and the triple injection model-based AIF (1). Tissue TACs were simulated using three values for each kinetic parameter, chosen to span a physiological meaningful range: $K_{1} = 0.05,{ }0.075,{ }0.1{ }$(ml/g/min), $k_{2} = 0.05,{ }0.15,{ }0.25$ (1/min), ${ }k_{3} = 0.02,{ }0.03,{ }0.04$ (1/min) and $v_{b}$ was fixed at 0.03. For each of the 27 TACs corresponding to each parameter combination, we generated 1000 TACs and added random noise using (2) with noise levels of *c* = 0.1 and *c* = 0.4, chosen to reflect human parametric PET data realistically.

We set injection times to $t_{1} = 0$, $t_{2} = 42.5$ and $t_{3} = 49$ min and evaluated two cases:

1. Kinetic parameter estimation using segments of the TAC between 36 and 39 min and 48–60 min and the AIF from 39 to 48 min, as proposed in the triple injection protocol.
2. Kinetic parameter estimation using the TAC between 48 and 60 min and the AIF from 39 to 48 min, to verify the need for the late imaging time window after the first injection.

The nonlinear least square method was used to estimate kinetic model parameters. We calculated the mean and SD of parameter estimates, the relative error between estimated parameters and ground truth and the coefficient of variation (${\text{CV}}_{P}$) for each parameter.

Figure 1c shows the corresponding TACs at two noise levels and mean, SD, relative error and ${\text{CV}}_{P}$ for parameter estimates at each noise level are provided in Additional file 1: Table S1. When 36- to 39-min and 48- to 60-min data were used, the mean relative error for all parameter estimates at both noise levels was less than ± 4% except for $k_{2}$ at high noise levels where the mean relative error was 8.8%. Omission of 36–39-min data resulted in larger mean error and ${\text{CV}}_{P}$ and a greater sensitivity to noise for estimates of individual parameters, with much less effect on estimates of $K_{i}$ (Additional file 1: Table S2).

## Methods

### Human PET imaging

#### Study participants

Approval for this project was granted by the Human Ethics Committee of the University of Queensland (2021/HE001605). Written informed consent was obtained from 6 healthy male adult participants (age 22–33, weight 45–95 kg). A summary of the participants' information is provided in Additional file 1: Table S1.

#### PET-CT data acquisition

**Fig. 3:**
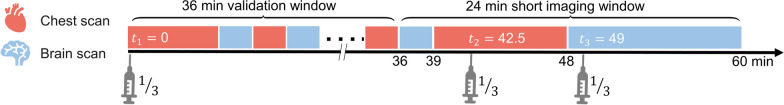
Schematic illustration of the protocol used for experimental validation of the triple injection method. Timings for the validation and short imaging windows and of the three tracer injections are depicted

Data were acquired on a Siemens Biograph Horizon PET scanner (Biograph Horizon 3R-VJ21C) at the Centre for Advanced Imaging, The University of Queensland. The acquisition protocol is summarised in Fig. 3 and differs from the triple injection protocol with the addition of dynamic PET data collection in the 36 min after the first tracer injection as a validation data set.

The total amount of ^18^F-FDG injected was approximately 200 MBq (total dose range: 181—203 MBq). Each of the three injections, comprising approximately one third of the total dose, was administered as an intravenous bolus followed by a 50 ml saline flush. List-mode acquisition started at the same time as the first injection of 18F-FDG. As shown in Fig. 3, the 60-min data acquisition consisted of a 36-min validation window (0–36 min) and the 24-min short imaging window (36–60 min). Image time frames comprising 3 × 20 s, 4 × 30 s, 3 × 60 s, 22 × 90 s, 3 × 60 s, 3 × 20 s, 4 × 30 s, 3 × 60 s, 3 × 60s, 2 × 90 s and 3 × 180 s were then reconstructed using TrueX + TOF (ultraHD-PET) with eight iterations and 20 subsets, a 2 mm Full Width at Half Maximum (FWHM) Gaussian filter was applied, along with a zoom factor of 2, resulting in images with a voxel size of 1.028 mm × 1.028 mm × 2.02 mm and a matrix size of 360 × 360. Random coincidence, scatter, attenuation, and radioactive decay corrections were performed. To mitigate the effects of noise, dynamic brain images were spatially filtered using a 2D Gaussian kernel in MATLAB® R2021b (MathWorks, Natick, MA) with the `imgaussfilt` function FWHM of 4.11mm. The ultimate spatial resolution, ${\text{FWHM}}$, results from a combination of factors: the native in-plane resolution (${\text{FWHM}}_{{1}}$ = 4.37 mm) [60], a contribution from reconstruction (${\text{FWHM}}_{{2}}$ = 2 mm), and an additional Gaussian filter (${\text{FWHM}}_{{3}}$ = 4.1 mm). These yields ${\text{FWHM}}$ = 6.4 mm using the formula $\sqrt {\left( {{\text{FWHM}}_{1} } \right)^{2} + \left( {{\text{FWHM}}_{2} } \right)^{2} + \left( {{\text{FWHM}}_{3} } \right)^{2} }$.

### Region of interests

We used a 10-mm spherical ROI over the LV in cardiac images, with a gap from the myocardium to minimize bias from myocardial activity spill-in when measuring the AIF [47].

Gray and white matter ROIs in the brain were manually segmented using the ROI freehand tool in MATLAB® R2021b. The corresponding TACs were extracted and used for kinetic parameters estimation (Additional file 2).

To validate the generated voxel-wise brain parametric maps, 14 ROIs comprising the caudate, cerebellum, anterior cingulate, posterior cingulate, hippocampus, insula, putamen, occipital, parietal, lingual, midtemporal, amygdala, thalamus and white matter available in the MNI AAL atlas [61] were segmented. The MNI T1 weighted MRI template was co-registered to the brain PET images (48–60-min averaged time frames) using SPM5 methodology in the Pmod software package (PMOD 4.302, PMOD Technologies, Zurich, Switzerland). The transformation matrix generated was used to transform the MNI AAL atlas to each subject’s native PET coordinate space.

### AIF estimation

Data from the 24-min short imaging window (39–48 min, see Fig. 3) were used to estimate the AIF. The four Feng function parameters ($A_{1}$, $A_{2}$, $\mu_{1}$ and $\mu_{2}$.) were estimated by fitting the triple injection model-based AIF (1) to the mean activity values the LV ROI in this imaging window. The AIF for the 60-min imaging period was generated using the estimated AIF parameters and (1) to compare the AIF measurement in the validation window (0–36 min).

### AIF evaluation

The AIF parameters estimated using data in the short imaging window were validated by using the parameters to generate an AIF for the 0 to 36-min validation window. These values were compared with measured values from a LV ROI using ${\text{AUC}}_{{{\text{error}}}}$ [3] and NRMSE [4].

### Kinetic parameter estimation in regions of interest

Data from the 24-min short imaging window (36–39 min and 48–60 min, see Fig. 3) were used to estimate the kinetic parameters. Using the estimated AIF based on data between 39 and 48 min, the 2TCM (see [5]) was fitted to TACs obtained by averaging the voxel values in each ROI in the 24-min short imaging window. $K_{1}$ (ml/g/min), $k_{2}$ (1/min) and $k_{3}$ (1/min) were estimated. We maintained a fixed blood volume fraction, $v_{b}$, of 0.03 in brain tissue [62, 63] and assumed its constancy throughout the entire brain [64, 65]. The net influx rate, $K_{i}$, was computed according to:

6 $$K_{i} = \frac{{K_{1} k_{3} }}{{\left( {k_{2} + k_{3} { }} \right)}}{ }\left( {{\text{ml}}/{\text{g}}/{\text{min}}} \right).$$

### Kinetic parameter estimation in voxels

Voxel-wise brain parametric maps for $K_{1}$, $k_{2}$ and $k_{3}$ were generated using nonlinear least squares fitting. The TACs for each voxel from the 24-min short imaging window were extracted. The estimated AIF from data between 39 and 48 min was used as the input function, $v_{b}$ was fixed to 0.03, and $K_{i}$ for each voxel was also computed from the estimated kinetic parameters (refer to [6]).

### Kinetic model fitting

The MATLAB® function lsqcurvefit was used to fit the AIF function with $A_{1}$ constrained to 300—800 kBq/ml using the Levenberg–Marquardt algorithm. The same optimization algorithm was used to fit the 2TCM to ROI- and voxel-based TACs. Linear least squares (MATLAB function lsqlin) was used to estimate $K_{i}$ by Patlak analysis.

### Validation of kinetic parameters

The estimated $K_{1}$, $k_{2}$, $k_{3} ,$ and $K_{i}$ from the averaged activity curves over gray matter and white matter ROIs, and the averaged kinetic parameters over 14 ROIs, gray matter ROI (from manual segmentation) and the whole brain from the generated voxel-wise parametric maps were presented as mean ± SD and compared with previously published values.

For further empirical validation, the parameter estimates from the short imaging window were used to compute $K_{i}$ and this value was compared with the $K_{i}$ estimate obtained from the validation window using Patlak analysis and the measured LV-derived AIF (see Fig. 3). Patlak analysis was performed using 15- to 36-min data from the validation window to ensure that pseudo-equilibrium had been attained.

This validation was performed for average kinetic parameter estimates in ROIs and for voxel-wise parameter estimates.

We calculated the error between the $K_{i}$ values estimated from the 24-min short imaging window and the validation window. The Pearson correlation coefficient ($r$) between the two estimated $K_{i}$ values was calculated for gray matter and white matter voxels separately. $r$ between the averaged $K_{i}$ values from the short imaging and the validation window in the 14 ROIs was also calculated.

## Results

### AIF estimation

**Fig. 4:**
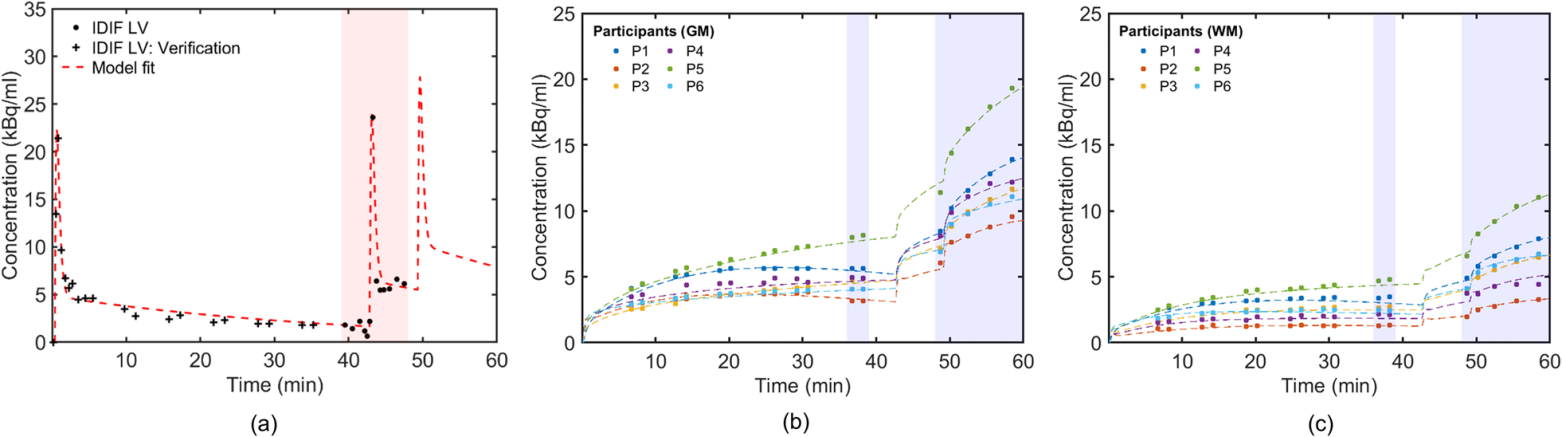
**a** Illustration of the estimated AIF for participant 6, **b** extrapolated gray matter and **c** white matter TACs for the six participants (P1-P6). **a** Data points denoted by the ‘ + ’ and ‘●’ symbols are measurements taken from the LV in the validation and the 39-48-min windows, respectively. Measurements from the 39-48min window (red shaded area) were used to estimate the entire AIF (the red dashed line). **b–c** The TAC measurements averaged over gray and white matter ROIs for the six participants (P1-P6). Data from 36-60min (blue shaded area) were used to estimate the kinetic parameters and recover the entire TAC (red dashed line). Measurements outside the 36-60min window were used for validation

Figure 4a shows the LV-derived AIF for Participant 6. The estimated AIFs for all participants captured the shape of the measured AIF in the validation window.

Table 2 summarizes the ${\text{AUC}}_{{{\text{error}}}}$ and NRMSE for each participant. The average ${\text{AUC}}_{{{\text{error}}}}$ and NRMSE across participants were 9.10% and 0.26, respectively. Estimates for the four Feng function parameters $A_{1}$, $A_{2} ,{ }\mu_{1}$ and $\mu_{2}$ for each participant are provided in Supplementary Material, Table 2.

**Table 2:** ${\text{AUC}}_{{{\text{error}}}}$ and NRMSE for the six participants’ LV derived arterial input function

Participants	AIF accuracy	
	${\text{AUC}}_{{{\text{error}}}}$ (%)	*NRMSE*
*P*1	3.35	0.11
*P*2	9.23	0.23
*P*3	14.72	0.38
*P*4	10.37	0.40
*P*5	6.33	0.28
*P*6	10.60	0.24
Mean ± *SD*	9.10 ± 3.90	0.26 ± 0.09

### Kinetic parameters estimation in region of interests

Figure 4b and Fig. 4c shows gray and white matter TACs for the six participants (P1-P6). In general, there was good agreement between TACs extrapolated into the validation window and measured TACs. Table 3 provides fitted model parameters. $K_{1}$, $k_{2}$, and $k_{3}$ estimates for gray matter were 0.08 ± 0.02 ${\text{ml/g/min}}$, 0.11 ± 0.03 and 0.04 ± 0.01 $1/min{ }$, respectively, clearly different from those of white matter which were, respectively, 0.05 ± 0.01 ${\text{ml/g/min}}$, 0.07 ± 0.02 and 0.03 ± 0.01 $1/{\text{min}}$. These estimates are within ranges published previously [66, 67]. Values of $K_{1}$ = 0.068–0.161 ${\text{ml/g/min}}$, $k_{2}$ = 0.070–0.301 $1/{\text{min}}$, $k_{3}$ = 0.030–0.098 $1/{\text{min}}$ have been reported [66] for gray matter using the 2TCM and arterial blood sampling. Previously reported kinetic parameters for white matter were $K_{1}$ = 0.047 ± 0.003 ${\text{ml/g/min}}$, $k_{2}$ = 0.07 ± 0.015 $1/{\text{min}}$, $k_{3}$ = 0.035 ± 0.005 $1/{\text{min}}$ [67].

**Table 3:** Kinetic parameters estimates from gray and white matter

	2TCM (short window)								Patlak (validation window)		Relative error (%) in ${\boldsymbol{K}}_{i}$ (short window against validation window)	
	${\boldsymbol{K}_{1}}$(ml/g/min)		${\boldsymbol{k}}_{2}$(1/min)		${\boldsymbol{k}}_{3}$(1/min)		${\boldsymbol{K}}_{i}$(ml/g/min)		${\boldsymbol{K}}_{i}$(ml/g/min)			
	GM	WM	GM	WM	GM	WM	GM	WM	GM	WM	GM	WM
P1	0.07	0.04	0.10	0.06	0.05	0.03	0.025	0.014	0.024	0.015	3.1	− 4.4
P2	0.06	0.03	0.08	0.05	0.03	0.02	0.015	0.007	0.016	0.010	− 1.1	− 26.0
P3	0.09	0.05	0.13	0.07	0.05	0.01	0.022	0.006	0.021	0.011	6.4	− 44.3
P4	0.07	0.04	0.10	0.06	0.05	0.03	0.025	0.014	0.024	0.015	3.1	− 4.4
P5	0.06	0.05	0.12	0.07	0.02	0.04	0.010	0.016	0.011	0.018	− 7.2	− 11.0
P6	0.10	0.06	0.15	0.10	0.04	0.02	0.021	0.012	0.020	0.014	7.0	− 13.6
Mean ± SD	0.08 ± 0.02	0.05 ± 0.01	0.11 ± 0.03	0.07 ± 0.02	0.04 ± 0.01	0.03 ± 0.01	0.020 ± 0.006	0.012 ± 0.004	0.019 ± 0.005	0.014 ± 0.003	1.9 ± 5.3	− 17.3 ± 15.4

Table 3 provides the estimated $K_{i}$ values from the validation window (0–36 min) and the relative error of the $K_{i}$ estimated from the 24-min shortened imaging window (36–60 min). Mean $K_{i}$ values in the 24-min short imaging window for gray and white matter were 0.02 ± 0.006 and 0.012 ± 0.004 ${\text{ml/g/min}}$, in close agreement with the validation window results of 0.019 ± 0.005 and 0.014 ± 0.003 ${\text{ml/g/min}}$. Across participants, the averaged $K_{i}$ value was underestimated by around − 17% for white matter and overestimated by less than 2% for gray matter.

### Voxel-wise parametric mapping

**Fig. 5:**
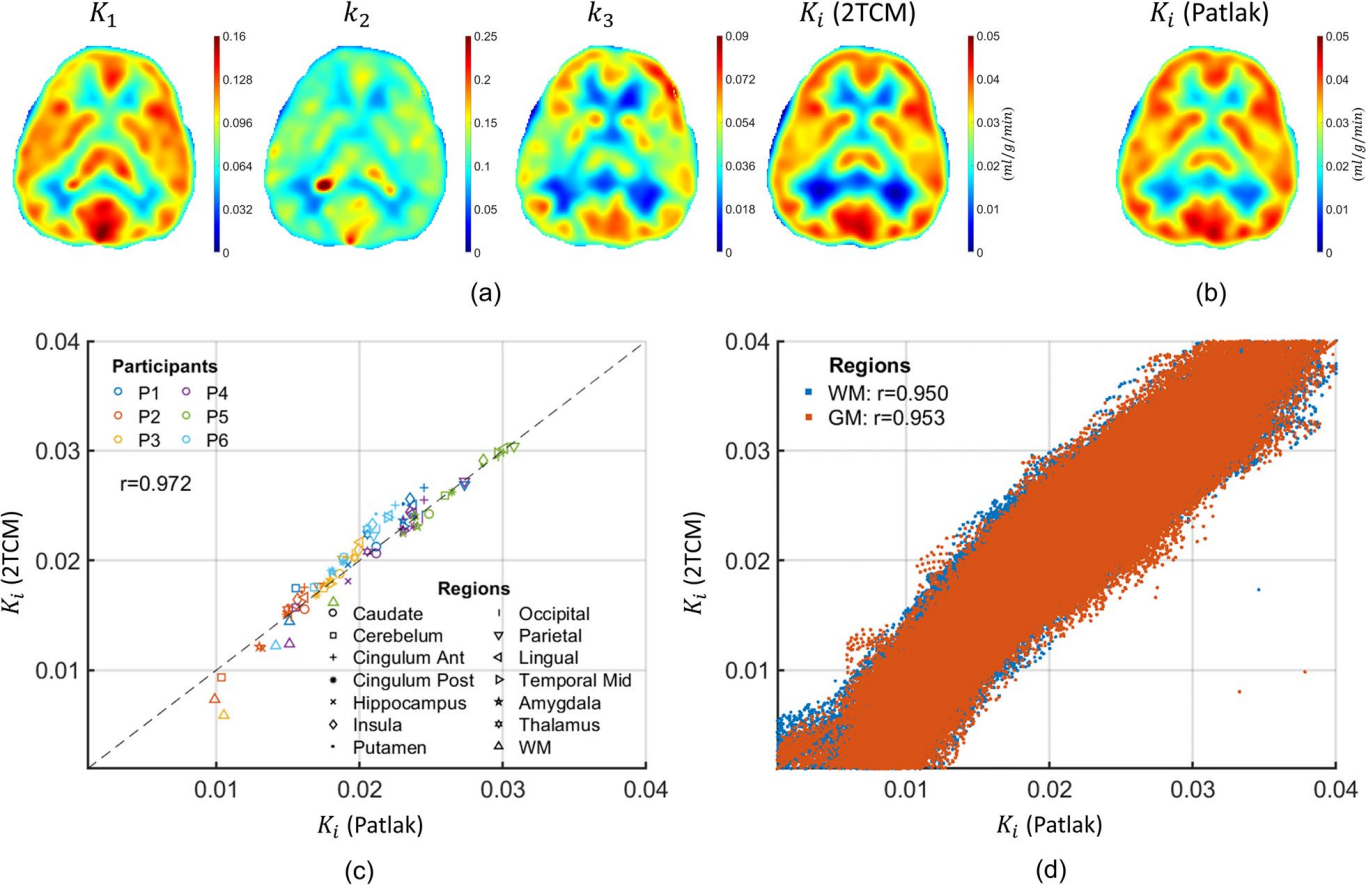
**a** Illustration of axial parameter maps of $K_{1}$(${\text{ml/g/min}}$), $k_{2} { }\left( {1/{\text{min}}} \right)$, $k_{3} { }\left( {\text{1/min}} \right)$, and $K_{i}$ (${\text{ml/g/min}}$) from Participant 5 produced using the 24-min short imaging window. **b** Map of $K_{i}$ (${\text{ml/g/min}}$) obtained using Patlak analysis of validation window data (0–36 min). **c** and **d**, respectively, depict plots of regional mean $K_{i}$ values and voxel-wise $K_{i}$ values determined using the 2TCM for short imaging window data and Patlak analysis for validation imaging window data in the six participants (P1–P6). **c** The brain $K_{i}$ maps were segmented into 14 primary brain regions including the caudate, cerebellum, anterior cingulate, posterior cingulate, frontal, hippocampus, insula, putamen, occipital, parietal, lingual, midtemporal, medial temporal, thalamus and white matter, available in the MNI AAL atlas. $K_{i}$ values were averaged over these ROIs. **d** Voxel-wise scatter plot. The Pearson correlation coefficient ($r$) was calculated between the $K_{i}$ estimated from the short imaging and validation windows

Figure 5 summarizes the voxel-wise parametric mapping results. Figure 5a depicts maps of $K_{1}$, $k_{2}$, $k_{3}$ and $K_{i}$ in one example slice in the brain region for Participant 5 produced using 2TCM from the 24 min short window data. Figure 5b illustrates the $K_{i}$ map generated using the Patlak analysis from the validation window. Qualitatively, the 24-min window parametric maps did not appear ‘noisy.’ The plots in Fig. 5c and Fig. 5d show the relationship between the mean $K_{i}$ values generated using the 2TCM from the 24-min short imaging widow and using Patlak analysis in the validation window in ROIs as well as the relationship at voxel level, separately for gray matter and white matter voxels. A strong correlation was found between the two $K_{i}$ estimates (ROIs: $r =$ 0.97, voxel-level gray matter: $r =$ 0.95 and voxel-level white matter: $r =$ 0.95).

Table 4 provides the estimated individual parameters ($K_{1}$, $k_{2}$, $k_{3}$, and $K_{i}$) for the 14 ROIs, the gray matter ROI and whole brain, and the estimated $K_{i}$ values using Patlak analysis from the validation window are provided for comparison. The highest $K_{1}$ and $k_{2}$ values were in the cerebellum (0.1 ± 0.04 ${\text{ml/g/min}}$ and 0.16 ± 0.07 $1/{\text{min}}$). These findings agree with previously reported largest values associated with the cerebellum, $K_{1}$ = 0.101–0.13 ${\text{ml/g/min}}$ and $k_{2}$ = 0.14–0.62 $1/{\text{min}}$ [68]. The highest $k_{3}$ and $K_{i}$ values were in the anterior cingulate, parietal, putamen and insula around $k_{3} =$ 0.05 $1/{\text{min}}$ and $K_{i} =$ 0.025 $1/{\text{min}}$. These values are within the range of the previously reported $k_{3} =$ 0.035–0.061 $1/min$ and $K_{i} =$ 0.016–0.03 ${\text{ml/g/min}}$ in the normal human brain, with the largest $k_{3}$ and $K_{i}$ found in the thalamus, parietal, cingulate and insular cortex [69].

**Table 4:** Kinetic parameters estimates from 14 ROIs, gray and white matter and whole brain

	2TCM (24 min short window)				Patlak (validation window) $K_{i}$(ml/g/min)	Relative error (%) in $K_{i}$ (short window against validation window)
Region	$K_{1}$(ml/g/min)	$k_{2}$(1/min)	$k_{3}$(1/min)	$K_{i}$(ml/g/min)		
Caudate	0.07 ± 0.02	0.11 ± 0.04	0.04 ± 0.02	0.020 ± 0.007	0.020 ± 0.006	0.5 ± 3.8
Cerebellum	0.10 ± 0.04	0.16 ± 0.07	0.04 ± 0.02	0.018 ± 0.007	0.017 ± 0.005	3.3 ± 8.5
Cingulum Ant	0.08 ± 0.03	0.12 ± 0.04	0.05 ± 0.02	0.025 ± 0.008	0.023 ± 0.006	7.2 ± 4.2
Cingulum Post	0.08 ± 0.03	0.12 ± 0.05	0.05 ± 0.02	0.021 ± 0.009	0.021 ± 0.008	1.3 ± 2.4
Hippocampus	0.08 ± 0.02	0.12 ± 0.04	0.04 ± 0.02	0.018 ± 0.007	0.018 ± 0.005	− 0.2 ± 5.3
Insula	0.08 ± 0.02	0.12 ± 0.04	0.05 ± 0.02	0.024 ± 0.007	0.022 ± 0.005	6.9 ± 3.6
Putamen	0.08 ± 0.02	0.12 ± 0.04	0.05 ± 0.01	0.023 ± 0.007	0.022 ± 0.005	8.0 ± 4.6
Occipital	0.08 ± 0.03	0.12 ± 0.04	0.05 ± 0.02	0.023 ± 0.008	0.023 ± 0.006	1.3 ± 4.1
Parietal	0.08 ± 0.02	0.1 ± 0.03	0.05 ± 0.02	0.024 ± 0.007	0.024 ± 0.006	2.0 ± 4.0
Lingual	0.09 ± 0.03	0.13 ± 0.05	0.05 ± 0.02	0.024 ± 0.007	0.023 ± 0.006	5.3 ± 3.3
Temporal Mid	0.08 ± 0.02	0.11 ± 0.04	0.05 ± 0.02	0.022 ± 0.008	0.022 ± 0.006	2.0 ± 3.6
Amygdala	0.08 ± 0.03	0.12 ± 0.06	0.04 ± 0.02	0.020 ± 0.008	0.020 ± 0.007	0.2 ± 4.5
Thalamus	0.08 ± 0.02	0.11 ± 0.04	0.05 ± 0.01	0.021 ± 0.006	0.020 ± 0.005	6.3 ± 4.2
White matter	0.05 ± 0.01	0.07 ± 0.02	0.03 ± 0.01	0.012 ± 0.004	0.014 ± 0.003	− 17.3 ± 15.4
Gray Matter	0.08 ± 0.03	0.11 ± 0.04	0.04 ± 0.02	0.021 ± 0.008	0.021 ± 0.006	2.0 ± 5.0
Whole Brain (mean)	0.08 ± 0.02	0.12 ± 0.04	0.04 ± 0.02	0.021 ± 0.007	0.021 ± 0.006	1.9 ± 5.1

Table 4 summarizes the average $K_{i}$ estimation error between the 24-min window kinetic parameters and the validation values. The greatest over estimation was reported in the putamen, 8%, while the greatest underestimation was found in the white matter, − 17.3%. In general, the $K_{i}$ estimation in the gray matter (errors = 2.0 ± 5.0%) was more accurate than white matter (errors = − 17.3 ± 15.4%).

## Discussion

We propose a triple injection protocol (see Fig. 1) that enables parametric brain imaging with ^18^F-FDG in a short (24 min) imaging window, using standard field of view PET scanners and an AIF noninvasively estimated from cardiac images.

Our simulation studies predicted that the AIF can be reliably estimated using only 9 min of cardiac imaging and a model-based input function. They also predicted that kinetic parameters can be estimated accurately using information from tissue TACs between 36–39 min and 48–60 min of the triple injection PET imaging protocol. TAC data from 36–39-min stabilized parameters estimates using the 2TCM.

The triple injection protocol was validated using dynamic PET images from 6 participants. The AIF estimated using only 9 min (39–48 min) of left ventricle measurements achieved a mean ${\text{AUC}}_{{{\text{error}}}}$ of 9% when compared with the AIF measured in the validation window.

Voxel-wise parametric mapping was successfully applied to participants’ short imaging window PET data (see Fig. 5a and Fig. 5b). Estimated kinetic parameters were in line with those expected for different regions in the brain [67, 68]. We observed disparities in mean values compared to published studies that employed high-resolution and high-sensitivity systems [40, 56, 70]. These variations are likely due to the lower spatial resolution scanner, reduced radiotracer dosages, and smoothing used in our study.

We also found a strong correlation between the voxel-wise $K_{i}$ values determined using parameter estimates from the 2TCM in the short imaging window and values obtained by Patlak analysis in the validation window ($r > 0.95$). In general, the stability and accuracy of $K_{i}$ estimates were greater in gray matter than in white matter. The larger variability in $K_{i}$ in white matter may relate to the effects of a higher noise in low activity regions on parameter estimates using the 2TCM [67].

Previous studies have reported that $K_{i}$ estimates from the Patlak analysis and 2TCM are highly correlated but with biases related to the time from which pseudo-equilibrium is assumed for Patlak analysis [71]. In our study, Patlak analysis was performed using the 36-min data acquisition after the first injection in the validation window. This is comparable to the ~ 40-min imaging time window used in a previous study, which consisted of 6 min at the cardiac bed position followed by dynamic scanning of the whole-body [59].

### Short window dynamic PET acquisitions

Double imaging window dynamic PET acquisitions have been previously proposed in which an initial 10- to 15-min scan is followed by 40-min rest before a 5-min scan with a total scan time of around 1 h [53–55]. Viswanath et al. evaluated two protocols using ^18^F-FDG and a long axial field-of-view PET scanner [54] and found that an early imaging window (0 to 10–15 min) followed by a scan at 60–65 min led to less than 10% bias in estimates of $K_{i}$ and $K_{1}$. In our study estimation of kinetic parameters using an early (0 to 12 min) and a late (57 to 60 min) window achieved an $R^{2}$ of 0.91 but $R^{2}$ decreased to 0.51 when the late window was omitted [52, 72].

Despite a reduction in acquisition time, previously proposed double imaging window methods are not clinically feasible because the participant is required to have two separate PET and CT scans, necessitating PET image co-registration. This motivated us to combine the two key imaging windows into one dynamic PET data acquisition, by appending early time point images after late time point images (see Fig. 1). An overall imaging session of around 24 min and three individual tracer injections were required to enable image-derived estimates of the AIF.

### Arterial input function estimation

Our PET protocol is designed to allow images of the heart to be used for noninvasive estimation of the AIF in standard field of view scanners by moving the scanner bed. This approach is clinically feasible, and enables $K_{1}$, $k_{2}$, $k_{3}$ and $K_{i}$ to be mapped. Previous approaches which derive the AIF from a region of interest over the left ventricle using a ~ 6-min acquisition over the chest followed by dynamic scanning of the whole-body using multi-bed passes including chest passes are sufficient for Patlak analysis [59] but the first 6 min of brain tissue TACs are required for accurate estimation of $K_{1}$, $k_{2}$, and $k_{3}$. Our triple injection protocol allows accurate left ventricle derived AIF estimation from 9-min dynamic PET data acquired over heart (red area in Fig. 4a). The 3 min of dynamic images over the heart provide the AIF ‘tail’ from the first injection and the last 6 min, acquired after the second injection, captures the initial part of the AIF including its peak. Segments of the tissue TAC between 0 and 12 min and 57–60 min predicted by simulations to be important for accurate parameter estimation were appended to the 9-min chest acquisitions, resulting in a 24-min acquisition incorporating bed movement. The short imaging window PET protocol was able to generate $K_{1}$, $k_{2}$ and $k_{3}$ estimates as well as $K_{i}$ estimates which compared well against $K_{i}$ obtained using Patlak analysis of data from the validation window.

Wu et al. recently described a double injection protocol using a total body PET (uEXPLORER system). The protocol included a tracer injection at the beginning of experiment and a single imaging window between 50 and 60 min, during which a second injection was administered at 56 min [56]. A combination of population-based and model-based assumptions were employed in AIF estimation. Wu et al.’s method [56] only yields $K_{i}$ estimates with the estimation error being less than 2%, comparable to our error. Our protocol was designed for standard field of view scanners which currently have a much larger installed base than long field-of-view (total body) scanners.

In our study, dividing the standard radiotracer dosage into three injections may have compromised image quality with shorter frame durations. We opted for a 20-s frame duration due to concerns about noise levels in images with shorter frame durations. The 20-s frames might not accurately capture the AIF peak, potentially introducing bias. Future studies could evaluate dosage adjustments and recently launched high-sensitivity scanners [40, 45, 46, 56] may also enable shorter frame intervals to more accurately capture the AIF peak.

Although there is potential for variability in injection rate due to manual administration of the three doses, diligent efforts were made to ensure consistency in the injection profiles. Implementing an injector system in future studies could enhance precision and reduce this variability.

We chose a third-order Feng model over the fourth-order Feng model [57] due to our three-injection equation's complexity, which involved convolving three distinct impulse functions with the Feng model. Fitting the convolved fourth-order model to the short window AIF measurement was not feasible without extensive manual adjustment and strict parameter constraints. Hence, we opted to use the third-order model which did not require these manipulations. Feng et al. [57] explored both third- and fourth-order models and found no significant disparity in kinetic parameter estimation, especially with noisy data. Given our study's short AIF measurements and lower radiotracer doses, which increased noise levels, we considered the third-order Feng model to be a reasonable choice.

Although our protocol was validated for use in brain imaging, there is no reason why this method cannot be adapted for parametric imaging of other body parts and organs. The requirement for three separate injections is only likely to be a minor limitation because the same cannula is used. However, clinical acceptance and practical utility of the protocol remains to be evaluated. In addition, the effects of varying the three injected doses should be evaluated as this may be a consideration in clinical implementation.

### Future directions

Our study only involved healthy participants and future work should investigate applications in a clinical cohort, such as oncology patients, and evaluate patient acceptance and impact on imaging throughput. A similar protocol may also be suitable for parametric imaging of other tracers.

## Conclusion

Our triple injection protocol enables parametric ^18^F-FDG imaging, with noninvasive estimation of the AIF, from a single 24-min imaging window using a standard field of view PET scanner.

### Supplementary Information

**Additional file 1. Table S1**: Kinetic parameters statistics obtained from simulation results for a 24 minute window, including initial brain scan. **Table S2:** Kinetic parameters statistics obtained from simulation results for a 21 minute window, excluding initial brain scan.

**Additional file 2. Table 1: **Summery of participants information. **Table S2:** Estimates for the Feng function parameters A_1, A_2,μ_1 and μ_2 for estimated participants AIF (P1-P6).

## Data Availability

The datasets generated during and/or analyzed during the current study are available from the corresponding author on reasonable request.

## Abbreviations

PET: Positron emission tomography
SUV: Standardized uptake value
AIF: Arterial input function
ROI: Regions of interest
TAC: Time activity curve
SD: Standard deviation
AUC: Area under the curve
NRMSE: Normalized root-mean-square error
2TCM: Two-tissue compartment model
LV: Left ventricle
