# Supplementary material for: A short 18F-FDG imaging window triple injection neuroimaging protocol for parametric mapping in PET
Source: EJNMMI Res. 2024 Jan 2;14:1. doi: 10.1186/s13550-023-01061-7 (PMC10761663; doi:10.1186/s13550-023-01061-7)
Supplement: Supplementary file 2 — Additional file 2. Table 1: Summery of participants information. Table S2: Estimates for the Feng function parameters A_1, A_2,μ_1 and μ_2 for estimated participants AIF (P1-P6). [file 13550_2023_1061_MOESM2_ESM.docx]

**A short ^18^F-FDG imaging window triple injection neuroimaging protocol for parametric mapping in PET**

Hamed Moradi,**^1, 2^** Rajat Vashistha,**^1, 2^** Kieran O'Brien,**^1, 2, 3^** Amanda Hammond,**^2, 3^** Viktor Vegh,**^1, 2^** David Reutens,**^1,2^**

^1^Centre for Advanced Imaging, [Australian Institute for Bioengineering and Nanotechnology](https://aibn.uq.edu.au/), The University of Queensland, Brisbane, Australia

^2^ARC Training Centre for Innovation in Biomedical Imaging Technology, The University of Queensland, Brisbane, Australia

^3^Siemens Healthcare Pty Ltd, Melbourne, Australia

**Corresponding Author:** Viktor Vegh

Centre for Advanced Imaging

[Australian Institute for Bioengineering and Nanotechnology](https://aibn.uq.edu.au/)

The University of Queensland

Brisbane, Australia

Email: [v.vegh@uq.edu.au](mailto:v.vegh@uq.edu.au), [viktor.vegh@cai.uq.edu.au](mailto:viktor.vegh@cai.uq.edu.au)

Table 1: Summery of participants information

| **Participants** | **Sex** | **Age** | **Weight (kg)** | **Total injected dose (MBq)** |
| --- | --- | --- | --- | --- |
| **P1** | M | 31 | 60 | 189.23 |
| **P2** | M | 22 | 88 | 180.92 |
| **P3** | M | 26 | 80 | 203.53 |
| **P4** | M | 28 | 80 | 202.32 |
| **P5** | M | 29 | 45 | 194.73 |
| **P6** | M | 33 | 95 | 202.96 |

Table 2: Estimates for the Feng function parameters $A_{1}$, $A_{2}, \mu_{1}$ and $\mu_{2}$ for estimated participants AIF (P1-P6)

| **Participants** | **AIF parameters** | | | |
| --- | --- | --- | --- | --- |
|  | $\boldsymbol{A}_{\mathbf{1}}$ **(kBq/ml)** | $\boldsymbol{A}_{\mathbf{2}}$ **(kBq/ml)** | $\boldsymbol{\mu}_{\mathbf{1}}$ **(1/min)** | $\boldsymbol{\mu}_{\mathbf{2}}$ **(1/min)** |
| **P1** | 322 | 16.2 | 2.7 | 0.023 |
| **P2** | 770 | 12.6 | 4 | 0.031 |
| **P3** | 455 | 15.1 | 3.9 | 0.021 |
| **P4** | 545 | 15 | 4.7 | 0.05 |
| **P5** | 606 | 23.4 | 4.1 | 0.028 |
| **P6** | 641 | 14.6 | 4 | 0.025 |
| **Mean ± SD** | 557±155 | 16.2±3.8 | 3.9±0.7 | 0.030±0.011 |
